# Modeling Interventions to Reduce the Spread of Multidrug-Resistant Organisms Between Health Care Facilities in a Region

**DOI:** 10.1001/jamanetworkopen.2021.19212

**Published:** 2021-08-04

**Authors:** Sarah M. Bartsch, Kim F. Wong, Leslie E. Mueller, Gabrielle M. Gussin, James A. McKinnell, Thomas Tjoa, Patrick T. Wedlock, Jiayi He, Justin Chang, Shruti K. Gohil, Loren G. Miller, Susan S. Huang, Bruce Y. Lee

**Affiliations:** 1 Public Health Informatics, Computational, and Operations Research, Graduate School of Public Health and Health Policy, City University of New York, New York, New York; 2 Center for Simulation and Modeling, University of Pittsburgh, Pittsburgh, Pennsylvania; 3 Division of Infectious Diseases and Health Policy Research Institute, Health School of Medicine, University of California–Irvine, Irvine; 4 Infectious Disease Clinical Outcomes Research Unit, Lundquist Institute, Harbor-UCLA Medical Center, Torrance, California; 5 Torrance Memorial Medical Center, Torrance, California

## Abstract

**Importance:**

Multidrug-resistant organisms (MDROs) can spread across health care facilities in a region. Because of limited resources, certain interventions can be implemented in only some facilities; thus, decision-makers need to evaluate which interventions may be best to implement.

**Objective:**

To identify a group of target facilities and assess which MDRO intervention would be best to implement in the Shared Healthcare Intervention to Eliminate Life-threatening Dissemination of MDROs in Orange County, a large regional public health collaborative in Orange County, California.

**Design, Setting, and Participants:**

An agent-based model of health care facilities was developed in 2016 to simulate the spread of methicillin-resistant *Staphylococcus aureus* (MRSA) and carbapenem-resistant Enterobacteriaceae (CRE) for 10 years starting in 2010 and to simulate the use of various MDRO interventions for 3 years starting in 2017. All health care facilities (23 hospitals, 5 long-term acute care hospitals, and 74 nursing homes) serving adult inpatients in Orange County, California, were included, and 42 target facilities were identified via network analyses.

**Exposures:**

Increasing contact precaution effectiveness, increasing interfacility communication about patients’ MDRO status, and performing decolonization using antiseptic bathing soap and a nasal product in a specific group of target facilities.

**Main Outcomes and Measures:**

MRSA and CRE prevalence and number of new carriers (ie, transmission events).

**Results:**

Compared with continuing infection control measures used in Orange County as of 2017, increasing contact precaution effectiveness from 40% to 64% in 42 target facilities yielded relative reductions of 0.8% (range, 0.5%-1.1%) in MRSA prevalence and 2.4% (range, 0.8%-4.6%) in CRE prevalence in health care facilities countywide after 3 years, averting 761 new MRSA transmission events (95% CI, 756-765 events) and 166 new CRE transmission events (95% CI, 158-174 events). Increasing interfacility communication of patients’ MDRO status to 80% in these target facilities produced no changes in the prevalence or transmission of MRDOs. Implementing decolonization procedures (clearance probability: 39% in hospitals, 27% in long-term acute care facilities, and 3% in nursing homes) yielded a relative reduction of 23.7% (range, 23.5%-23.9%) in MRSA prevalence, averting 3515 new transmission events (95% CI, 3509-3521 events). Increasing the effectiveness of antiseptic bathing soap to 48% yielded a relative reduction of 39.9% (range, 38.5%-41.5%) in CRE prevalence, averting 1435 new transmission events (95% CI, 1427-1442 events).

**Conclusions and Relevance:**

The findings of this study highlight the ways in which modeling can inform design of regional interventions and suggested that decolonization would be the best strategy for the Shared Healthcare Intervention to Eliminate Life-threatening Dissemination of MDROs in Orange County.

## Key Points

**Question:**

Which multidrug-resistant organism (MDRO) intervention is best to implement in a set of health care facilities to reduce the spread of MDROs regionwide?

**Findings:**

In this computational simulation modeling study of 102 facilities in Orange County, California, the use of an agent-based model indicated that, after 3 years, increasing contact precaution effectiveness and implementing decolonization procedures in 42 target facilities would yield countywide relative decreases in the prevalence of methicillin-resistant *Staphylococcus aureus* of 1% and 24% and countywide relative decreases in the prevalence of carbapenem-resistant Enterobacteriaceae of 2% and 40%, respectively, but varied with effectiveness. Increasing interfacility communication produced no changes in prevalence or transmission.

**Meaning:**

This study’s findings suggest that modeling can inform the design of real-world regional interventions to control MDROs when intervening in a set of health care facilities.

## Teaser

This computational simulation modeling study uses an agent-based model to identify target facilities and assess the optimal intervention to reduce the regional spread of multidrug-resistant organisms across health care facilities in Orange County, California.

## Introduction

Multidrug-resistant organisms (MDROs) can spread across health care facilities in a region; when resources are limited and interventions can be implemented in only some facilities, decision-makers need to evaluate which interventions are best to implement. Previous work found that health care facilities in a region form a complex system and are highly interconnected by patient sharing^1,2^; thus, patients often carry MDROs from 1 facility to another. Furthermore, outbreaks of MDROs, such as methicillin-resistant *Staphylococcus aureus* (MRSA), vancomycin-resistant enterococci, and carbapenem-resistant Enterobacteriaceae (CRE) can occur across a region and can spread in unpredictable ways.^3,4,5,6,7^ In addition, various infection prevention and control measures implemented within and across different facilities affect each other in different ways, with varying indirect, synergistic, and reverberating consequences.^4,6,8,9,10^ These complexities make it difficult for decision-makers to identify the best interventions to implement and the optimal locations in which to intervene.

Computational models dynamically represent a system’s components and processes and serve as virtual laboratories to test the potential impact of different policies and interventions before real-world implementation, which can save time, effort, and resources. Moreover, models can help decision-makers identify the best combination of interventions to control multiple target pathogens (eg, MRSA and CRE) if only select facilities can implement interventions. The Shared Healthcare Intervention to Eliminate Life-threatening Dissemination of MDROs in Orange County (SHIELD-OC) was a regional public health intervention project funded by the Centers for Disease Control and Prevention.^11^ SHIELD-OC was designed to use modeling to identify target facilities and evaluate a high-yield MDRO prevention strategy for real-world implementation. In 2016, we developed an agent-based model of health care facilities in Orange County, California (using our Regional Healthcare Ecosystem Analyst software) to simulate the spread of MRSA and CRE over 10 years, starting in 2010, and the use of various MDRO interventions for 3 years, starting in 2017. We identified a group of target facilities and used the model to assess which prevention and control strategy would be best to implement in these facilities to reduce the spread of MRDOs regionwide.

## Methods

### Model of Orange County

As previously described,^3,12^ we used our Regional Healthcare Ecosystem Analyst software to generate an agent-based model of Orange County that represented the 23 hospitals, 5 long-term acute care hospitals, and 74 nursing homes serving adult inpatients in Orange County. The model simulated patients (each represented by a computational agent) moving in, out, and between each of the facilities (combined annual total of 240 955 hospital admissions, 705 long-term acute care hospital admissions, and 36 245 nursing home residents) and the spread of MRSA and CRE^3,4,5,6,8,9,10,12,13,14^ (eFigure 1 in the Supplement). Each facility was divided into various units. Each computational agent could carry either MRSA or CRE and transmit the pathogen to other agents in the same unit based on a formula in which the number of new daily carriers in each unit was calculated as the unit- and facility-specific transmission coefficient multiplied by the number of susceptible patients in that unit and by the number of infectious patients in that unit. The simulation proceeded via 1-day steps. Agents admitted to a facility drew from that facility’s length-of-stay distribution for a particular unit and stayed for that duration (additional details are available in eMethods in the Supplement). This study was approved by the Johns Hopkins Bloomberg School of Public Health institutional review board. The study used the Modeling Infectious Diseases in Healthcare Network (MIND-Healthcare) framework.^15^

### Choosing Target Facilities

The budget constraints of SHIELD-OC meant that approximately 40 facilities could participate in the project, half of which were nursing homes because nursing homes play a substantial role in the spread of MDROs among facilities in a region.^5^ Achieving the greatest result meant choosing facilities in which interventions could benefit as many patients as possible because the facilities are highly interconnected, and the extent of these connections increases the spread of MDROs to more facilities.^1,2,3,4,5^ We created a network map using 2011 to 2012 Orange County patient-level data,^16,17^ with nodes representing each facility. A directional line was drawn from facility A to facility B if facility A transferred, on average, at least 1 patient per week to facility B (with or without an intervening stay in the community). The number of patients transferred determined the line’s thickness. A line was similarly drawn from facility B to facility A if facility B transferred, on average, at least 1 patient per week to facility A. The out-degree for a given facility was defined as the number of patients who moved from that facility directly to other facilities or who were readmitted within 365 days; thus, out-degree was a measure of a facility’s volume of discharges transferred or readmitted when sending at least 1 patient per week. We ranked (highest to lowest) long-term acute care hospitals and nursing homes by total out-degree, and we ranked hospitals by out-degree to nursing homes because hospitals are highly interconnected with nursing homes.^2^ We then selected facilities with the highest out-degree from each list, excluding specialized facilities (eg, psychiatric).

### Choosing Potential Interventions

Using guidance for MDRO prevention strategies from the Society for Healthcare Epidemiology of America,^18^ we identified 3 interventions to evaluate in target facilities. The first intervention involved increasing contact precaution effectiveness. Placing an agent under contact precautions entailed reducing the agent’s probability of contact with other agents in the same unit by a certain percentage. Increasing contact precaution effectiveness by improving adherence meant increasing this probability from 40%^19,20,21,22,23^ to 48% and then from 40% to 64% for MRSA and CRE, respectively.

The second intervention involved increasing interfacility communication about MDRO status. This intervention entailed increasing the probability from 50% to 60% and then from 50% to 80% of a facility knowing whether an agent was colonized by MRSA or CRE before transfer from another facility; thus, this intervention was able to automatically place the agent under contact precautions. Because nursing homes only apply contact precautions to residents with clinically apparent infections,^24^ contact precautions were applied to known carriers on a 1:10 ratio (eg, 5% of known MRSA carriers and 10% of known CRE carriers); thus, increasing interfacility communication meant increasing from 5% to 6% and to 8% of known MRSA carriers and increasing from 10% to 12% and to 16% of known CRE carriers.

The third intervention involved performing decolonization using antiseptic bathing soap and a nasal product. In hospitals, decolonization consisted of ongoing daily chlorhexidine gluconate (CHG) bathing in intensive care units (ICUs), which is the current standard of care in the US, and the addition of CHG bathing and a nasal product (iodophor) for 5 days for MDRO carriers (identified as those under contact precautions in ICUs and non-ICUs). In long-term acute care hospitals, decolonization involved universal daily CHG bathing plus nasal iodophor for 5 days upon admission and Monday to Friday every other week thereafter. In nursing homes, decolonization involved CHG bathing 3 times per week plus nasal iodophor for 5 days upon admission and Monday to Friday every other week thereafter.

When a MRSA-colonized agent underwent decolonization, the agent had a probability of being decolonized (ie, changing from a MRSA carrier to a noncarrier) after 5 days, with an equal likelihood of clearing colonization on any of the 5 days. This probability varied by facility type. For agents in hospitals, the baseline probability was 39%, which represented the average clearance probability reported across studies evaluating the use of CHG bathing and a nasal product among hospitalized patients.^25,26,27,28,29^ Sensitivity analyses were used to explore the impact of varying the range of this probability from 24% to 54%. For agents in long-term acute care hospitals, the baseline probability was 27% (the lowest value reported among clinical trials of hospitalized patients^25^) because clearance among patients in long-term acute care hospitals may be more difficult owing to factors such as comorbidities, indwelling devices, wounds, and ventilator use.^29,30,31^ Because nasal decolonization occurred every other week, agents had this probability applied biweekly. Sensitivity analyses varied this probability from 18% to 42%. For agents in nursing homes, the baseline probability was lower at 3% because of differences in the quality and frequency of bathing (eg, lower staff to resident ratio or bathing 3 days per week) and resident characteristics (eg, comorbidities, indwelling devices, wounds, or bedbound status) that made it more difficult to clear colonization,^32^ and this probability was applied to each decolonization round (eg, every other week). Sensitivity analyses varied this probability from 1% to 16.7%. Agents who were successfully decolonized had a linear rate of MRSA relapse after discharge (changing colonization status at readmission); 20% of agents relapsed after 90 days,^26,29^ and 32% of agents relapsed after 240 days.^26,29,33^

Decolonization of CRE proceeded in a different manner because CRE carriage typically occurs in the gastrointestinal tract (rather than the skin and nose, as does MRSA); thus, CRE may not be cleared by topical decolonization. Instead, bathing may reduce transmission to or from that patient for the duration of bathing but not after bathing. Therefore, each day during the CHG bathing regimen, a CRE-colonized agent’s chances of transmitting CRE to other agents decreased by 48% (based on reduction in the incidence rate of CRE colonization in long-term acute care hospitals when using CHG bathing, which was reported in a multicenter intervention study^34^). If the agent was also under contact precautions, bathing decreased the chances of transmission by 60% (accounting for both interventions). Sensitivity analyses were used to explore varying this probability from 42% to 64% (52.5%-67.5% with contact precautions).

### Existing Situation in Orange County

Before implementing any potential SHIELD-OC intervention, we simulated existing circumstances in Orange County for the preceding 7 years (2010-2017). This included representation of interventions that Orange County facilities already had in place. All 28 acute care facilities actively screened patients for MRSA on ICU admission, direct transfer, and readmission within 30 days (consistent with California laws active since 2007). However, a facility only detected CRE carriage through cultures obtained for clinical reasons, thereby identifying only a small proportion of carriers (eTable 1 in the Supplement). Contact precautions were applied to those with a positive test result for MRSA (ie, true-positive and false-positive results, regardless of true colonization), those identified as CRE carriers, and those with a known history of MRSA or CRE carriage (including those readmitted to the same facility). Patients remained under contact precautions when transferred to other facilities based on the fidelity of interfacility communication (with a baseline of 50%). In all 74 nursing homes, contact precautions were only applied to those with clinically apparent MRSA and CRE infections^24^ (assumed to be 5% and 10% of known MRSA and CRE carriers, respectively, for 10 days^14^).

In addition, 19 Orange County acute care facilities already used daily CHG bathing. Of those, 4 facilities used universal hospital-wide CHG bathing, and 15 facilities used CHG bathing in the ICU; CHG bathing was implemented in 1 facility in 2008, 2 facilities in 2010, 3 facilities in 2011, 3 facilities in 2012, 2 facilities in 2013, and 8 facilities in 2014. In 2016, 1 facility switched from hospital-wide bathing to ICU bathing plus non-ICU device-related bathing (58% of patients). Thus, in our model, when a MRSA-colonized agent underwent CHG bathing, the agent had an 18% probability of clearing MRSA after 5 days (based on findings from a randomized clinical trial that evaluated the use of CHG bathing^35^). For a CRE-colonized agent, CHG bathing decreased the chances of transmitting CRE to and from other agents by 39% (based on findings from an intervention study and adjusted for lower adherence outside a research setting^34^) and by 48% for an agent under contact precautions.^34^ Different scenarios were then used to simulate the effects of implementing each of the 3 SHIELD-OC interventions, 1 intervention at a time, starting in April 2017 (day 2648), and assumed constant intervention effectiveness over a 3-year period.

## Results

### Selecting Target Facilities

We identified 42 target facilities: 18 hospitals (out-degree to nursing homes, 397-3357 patients; total out-degree, 2113-17 685 patients), 3 long-term acute care hospitals (total out-degree, 195-750 patients), and 21 nursing homes (total out-degree, 197-504 patients). The network map is shown in eFigure 2 in the Supplement.

### Continuing Existing Control Measures

Continuing existing control measures yielded a MRSA prevalence of 30.6% after 3 years, with a mean (SD) of 29 366 (8.8) new carriers across all health care facilities. Most transmission events occurred in nursing homes (23 646 new events). Continuing existing control measures yielded a CRE prevalence of 5.5% after 3 years, with a mean (SD) of 36 167 (15) new carriers countywide.

### Increasing Contact Precaution Effectiveness

**Figure 1.**
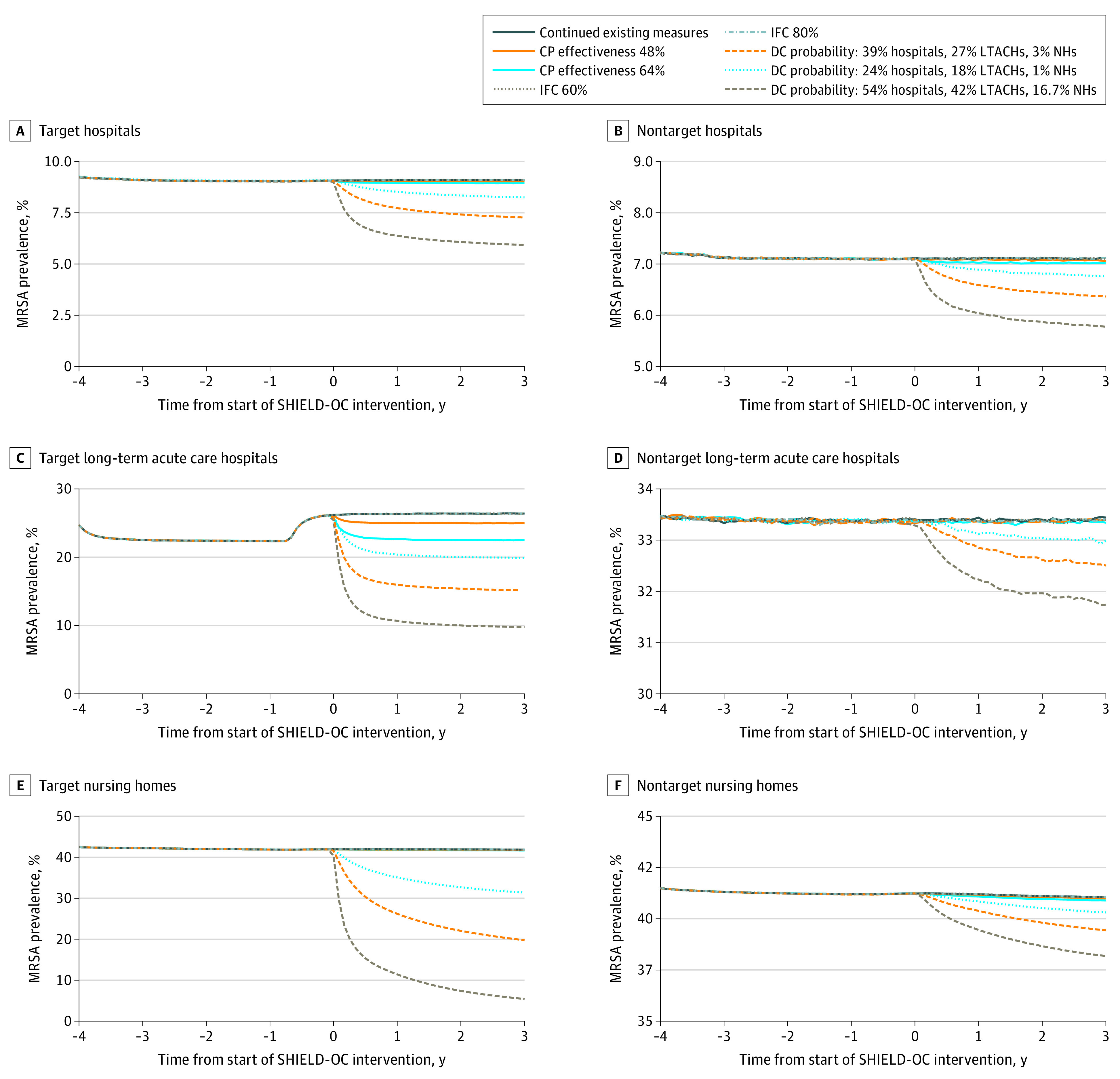
Prevalence of Methicillin-Resistant *Staphylococcus aureus *(MRSA) Over Time in Target and Nontarget Hospitals, Long-term Acute Care Hospitals, and Nursing Homes Implementing Potential Shared Healthcare Intervention to Eliminate Life-threatening Dissemination of MDROs in Orange County (SHIELD-OC) Interventions In panel C, the increase in prevalence is associated with 1 facility switching from hospital-wide bathing to intensive care unit and device-associated bathing (in 2016) before implementation of potential SHIELD-OC interventions. CP indicates contact precaution; DC, decolonization clearance; IFC, interfacility communication; LTACH, long-term acute care hospital; and NH, nursing home.

**Figure 2.**
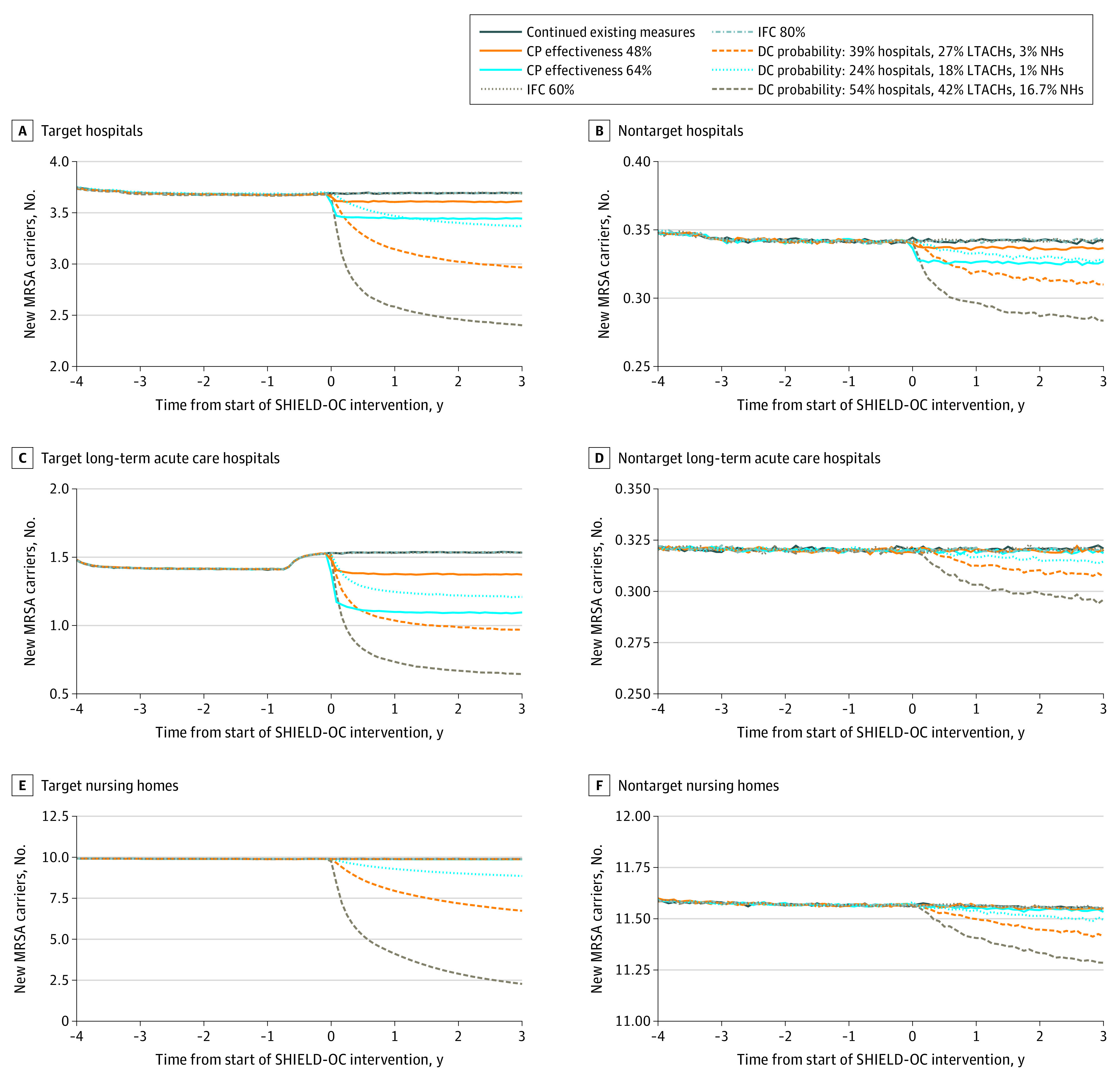
Daily Number of New Methicillin-Resistant *Staphylococcus aureus *(MRSA) Carriers in Target and Nontarget Hospitals, Long-term Acute Care Hospitals, and Nursing Homes Implementing Potential Shared Healthcare Intervention to Eliminate Life-threatening Dissemination of MDROs in Orange County (SHIELD-OC) Interventions In panel C, the increase in prevalence is associated with 1 facility switching from hospital-wide bathing to intensive care unit and device-associated bathing (in 2016) before implementation of potential SHIELD-OC interventions. CP indicates contact precaution; DC, decolonization clearance; IFC, interfacility communication; LTACH, long-term acute care hospital; and NH, nursing home.

Increasing contact precaution effectiveness from 40% to 48% and 64% yielded a relative reduction in MRSA prevalence of 0.3% (range, 0%-0.6%) and 0.8% (range, 0.5%-1.1%), respectively, in health care facilities countywide after 3 years. Long-term acute care hospitals experienced the largest reductions (eg, 14.6% relative reduction with 64% effectiveness) (Figure 1). Among target facilities, by year 3, MRSA prevalence decreased by a relative 0.6% or more in hospitals, 5.3% or more in long-term acute care hospitals, and 0.2% or more in nursing homes, with no reduction observed to a 0.8% relative reduction in nontarget facilities. Figure 2 shows daily new carriers by facility type in both target and nontarget facilities, and eTable 2 in the Supplement shows the cumulative number of new carriers over time. Countywide reductions accrued over time; after 3 years of increased effectiveness, there were 270 fewer transmission events (95% CI, 264-275 events) when effectiveness increased to 48% and 761 fewer transmission events (95% CI, 756-765 events) when effectiveness increased to 64% compared with the continuation of existing control measures. With regard to total new carriers, increasing contact precaution effectiveness to 48% and 64% yielded a relative decrease of 2.1% and 6.3% in hospitals, 8.4% and 22.9% in long-term acute care hospitals, and 0% and 0.1% in nursing homes, respectively.

**Figure 3.**
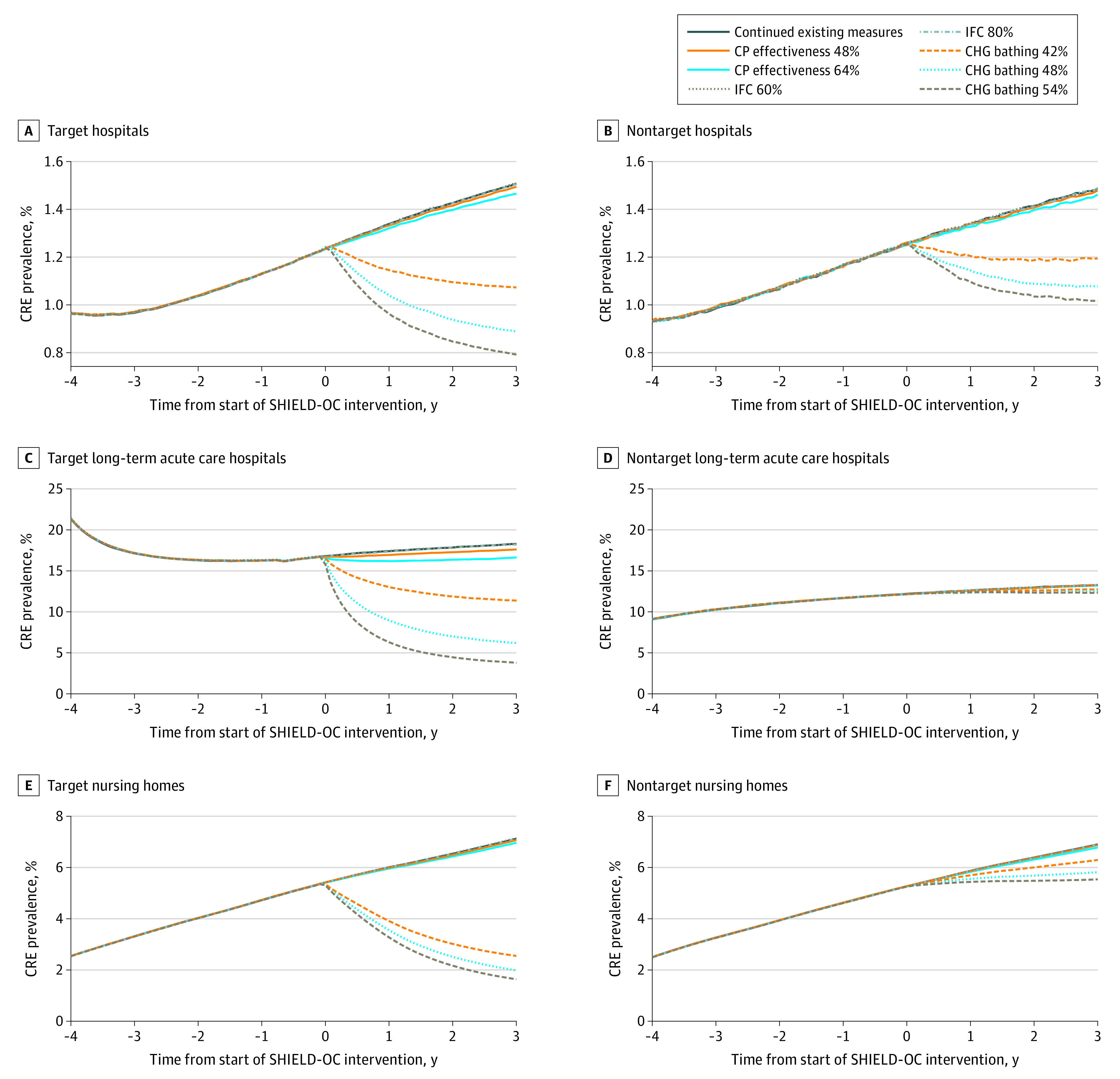
Prevalence of Carbapenem-Resistant Enterobacteriaceae (CRE) Over Time in Target and Nontarget Hospitals, Long-term Acute Care Hospitals, and Nursing Homes Implementing Potential Shared Healthcare Intervention to Eliminate Life-threatening Dissemination of MDROs in Orange County (SHIELD-OC) Interventions In panel C, the increase in prevalence is associated with 1 facility switching from hospital-wide bathing to intensive care unit and device-associated bathing (in 2016) before implementation of potential SHIELD-OC interventions. CHG indicates chlorhexidine gluconate; CP, contact precaution; and IFC, interfacility communication.

**Figure 4.**
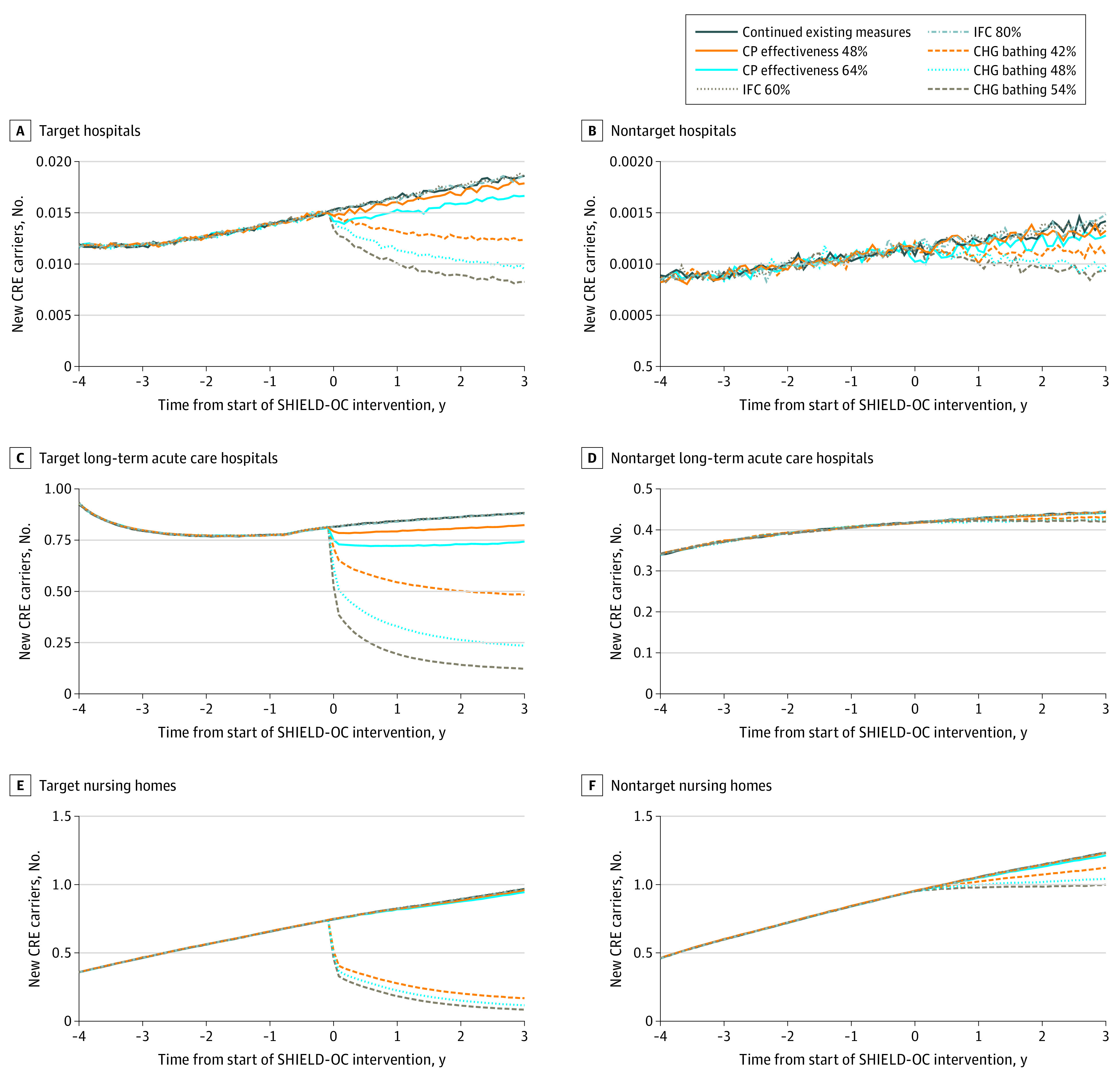
Daily Number of New Carbapenem-Resistant Enterobacteriaceae (CRE) Carriers in Target and Nontarget Hospitals, Long-term Acute Care Hospitals, and Nursing Homes Implementing Potential Shared Healthcare Intervention to Eliminate Life-threatening Dissemination of MDROs in Orange County (SHIELD-OC) Interventions In panel C, the increase in prevalence is associated with 1 facility switching from hospital-wide bathing to intensive care unit and device-associated bathing (in 2016) before implementation of potential SHIELD-OC interventions. CHG indicates chlorhexidine gluconate; CP, contact precaution; and IFC, interfacility communication.

Increasing adherence to contact precautions to 48% and 64% yielded a relative reduction in CRE prevalence of 0.8% (range, 0%-3.2%) and 2.4% (range, 0.8%-4.6%), respectively, in health care facilities countywide at 3 years. Among target facilities, at 48% and 64% effectiveness, the relative decrease in CRE prevalence by year 3 was 0.9% and 2.8% in hospitals, 3.6% and 9.0% in long-term acute care hospitals, and 0.7% and 2.2% in nursing homes (Figure 3). Increasing contact precaution effectiveness to 48% and 64% averted 64 new CRE transmission events (95% CI, 55-73 events) and 166 new CRE transmission events (95% CI, 158-174 events) countywide compared with continuing existing control measures. Among target facilities, increasing effectiveness to 48% and 64% yielded a relative decrease in total new carriers of 3.1% and 9.2% in hospitals, 5.9% and 14.5% in long-term acute care hospitals, and 0.5% and 1.5% in nursing homes after 3 years, respectively (Figure 4; eTable 3 in the Supplement).

### Increasing Interfacility Communication

Increasing interfacility communication of patients’ MDRO status produced no change in MRSA or CRE prevalence or transmission countywide. This result persisted even when communication increased to 80% (Figure 1 to Figure 4; eTable 2 and eTable 3 in the Supplement).

### Adding Chlorhexidine Gluconate Bathing

Implementing decolonization procedures for MRSA (clearance probability: 39% in hospitals, 27% in long-term acute care hospitals, and 3% in nursing homes) yielded a 23.7% (range, 23.5%-23.9%) relative reduction in prevalence in health care facilities countywide after 3 years. Among target facilities, the relative reduction in MRSA prevalence was 21.3% in hospitals, 42.6% in long-term acute care hospitals, and 52.9% in nursing homes. Benefits accrued quickly over the first 6 months and more slowly thereafter (Figure 1). Nontarget facilities also experienced reductions in MRSA prevalence and transmission (Figure 1 and Figure 2; eTable 2 in the Supplement). Among nontarget facilities, by year 3, the relative decrease in MRSA prevalence was 10.6% in hospitals, 2.8% in long-term acute care hospitals, and 3.9% in nursing homes. Decolonization averted 3515 transmission events (95% CI, 3509-3521 events) countywide, with 96% of those events averted in facilities that implemented decolonization (eTable 2 in the Supplement). Total transmission events decreased by a relative 14.3% in hospitals, 26.6% in long-term acute care hospitals, and 10.4% in nursing homes.

With a lower probability of clearance (24% in hospitals, 18% in long-term acute care hospitals, and 1% in nursing homes), decolonization yielded a relative reduction in MRSA prevalence of 11.2% (range, 11.0%-11.6%), averting 1326 transmission events (95% CI, 1321-1331 events) countywide after 3 years. Additional data regarding the impact of varying the decolonization clearance probability are shown in Figure 1, Figure 2, and eTable 2 in the Supplement.

With regard to CRE prevalence, 48% effectiveness in CHG bathing yielded a 39.9% (range, 38.5%-41.5%) relative decrease among health care facilities countywide after 3 years. Among target facilities, the relative decrease in CRE prevalence was 41.0% in hospitals, 66.1% in long-term acute care hospitals, and 70.1% in nursing homes (Figure 2). Among nontarget facilities, the relative decrease in CRE prevalence was 27.5% in hospitals, 5.9% in long-term acute care hospitals, and 15.8% in nursing homes. Increasing CHG bathing effectiveness to 48% averted 1435 total new transmission events (95% CI, 1427-1442 events) countywide, with 92% of those averted in target facilities (eTable 3 in the Supplement). Among target facilities, the relative decrease in CRE transmission events after 3 years was 34.4% in hospitals, 62.5% in long-term acute care hospitals, and 75.9% in nursing homes (Figure 4).

Decreasing CHG bathing effectiveness to 42% yielded a 31.0% (range, 29.7%-32.7%) relative reduction in CRE prevalence, averting 1807 new transmission events (95% CI, 1079-1906 events) countywide. Increasing effectiveness to 54% yielded a 45.0% (range, 44.0%-46.7%) relative reduction, averting 1643 new transmission events (95% CI, 1636-1651 events). Additional data regarding the impact of varying CHG bathing effectiveness in target and nontarget facilities are shown in Figure 3, Figure 4, and eTable 3 in the Supplement.

## Discussion

 The results of this computational simulation modeling study indicated that the best intervention option for SHIELD-OC was decolonization (ie, the use of CHG bathing and a nasal product), regardless of pathogen (MRSA or CRE). Decolonization yielded the largest reductions in prevalence and transmission; the benefits of decolonization took 6 months to manifest and continued to accrue over time, especially for MRSA. Although all interventions provided reductions in transmission, for MRSA, only decolonization produced a reduction in the carrier state itself. This finding is notable given the large proportion of patients in long-term acute care hospitals and nursing home residents who carry an MDRO (65%-80%^36^). In addition, decolonization is associated with reductions in MDRO shedding from the body,^37^ which not only reduces spread but may increase the margin of error for other infection prevention practices (eg, contact precautions and hand hygiene). Results were robust to changes in a carrier’s probability of being decolonized. Only when decolonization had a MRSA clearance probability of 24% in hospitals, 18% in long-term acute care hospitals, and 1% in nursing homes did increasing contact precaution effectiveness (to 64%) produce fewer new MRSA carriers in target hospitals and long-term acute care hospitals; this result was not observed in target nursing homes, nontarget facilities, and facilities countywide.

Compared with decolonization, contact precautions and interfacility communication of patients’ MDRO status have implementation barriers that could alter their value. For example, adherence to contact precautions among health care professionals is imperfect and may prevent contact precautions from having a greater benefit, particularly in nursing homes with guidelines requiring the use of contact precautions for infection rather than colonization.^24,38,39^ Although current electronic health systems allow seamless reinstitution of contact precautions at readmission to the same hospital,^40,41^ information relayed at transfer is variably used (eg, nursing homes typically only implement precautions for active infection). In addition, MDRO status is not commonly stored in an accessible location for nursing homes to transfer to other facilities, further limiting the value of interfacility communication.^42^ Although hospital admission screening for MRSA occurs by law in several states, other MDRO carriers must rely on interfacility communication to prompt the application of contact precautions at transfer.

The present study highlights the ways in which computational modeling can help to identify optimal MDRO prevention strategies. Because limited resources may restrict the number of facilities that are able to implement interventions, modeling can help decision-makers (eg, public health authorities, hospital administrators, and infection control practitioners) understand which interventions may yield the largest MRDO reductions regionwide. As the results of this and previous studies^6,7,10^ suggest, coordinating infection prevention efforts can maximize benefits for regional and individual facilities. However, the specific facilities targeted for interventions can alter the regional value.^43^ Thus, understanding patient-sharing patterns can help facilities coordinate to increase both direct and indirect benefits. Without this knowledge, a facility implementing highly successful interventions could garner benefits, but facilities that do not implement interventions may experience only limited benefits.^4,6,8,9,40,42^ In addition to reducing the burden of MDROs, costs are also an important consideration for intervention planning. Notably, the costs associated with MDRO infection^44,45,46^ are often substantially higher than the costs of interventions (eg, CHG wipes are approximately $5.50-$7.50 per bath,^47,48^ gloves and gowns are approximately $0.84 per use,^49^ and educational and printing materials for an intervention campaign are approximately $4606^49^). Furthermore, studies have reported that regional MDRO interventions (eg, registry, CHG bathing, and screening of interfacility transfers) are cost-saving.^43,50^

### Limitations

This study has limitations. Models, by definition, are simplifications of reality and cannot account for every possible outcome.^51^ We assumed the effectiveness and use of interventions were stable over time; however, adherence may vary across interventions. We included real-world decision-making by imposing constraints on the number of participating facilities in the analysis. Therefore, the best set of target facilities may not have been identified (eg, another set may have yielded greater reductions). Although changes in parameters, such as transmission coefficients, would alter MDRO spread, the changes would be similar across modeled scenarios and would not substantially change the resulting differences between them (eg, previous studies have found that increasing transmission coefficients, although increasing overall pathogen prevalence, produces similar outcomes when comparing scenarios^12,14^).

## Conclusions

This study highlights the ways in which modeling can inform the design of a real-world regional intervention by identifying the highest-yield intervention for multiple MDROs when intervening in a limited number of facilities. Decolonization provided the largest reductions in MDRO prevalence and spread, suggesting that this strategy would be the best option for SHIELD-OC.

## Notes

Supplement. **eMethods.** Description of Orange County, RHEA OC Software, and Model and Simulations **eTable 1.** MRSA and CRE Model Input Parameters, Values, and Sources **eTable 2.** Mean Number of New MRSA Carriers Over Time **eTable 3.** Mean Number of New CRE Carriers Over Time **eFigure 1.** Overview of the Structure of the Regional Healthcare Ecosystem Analyst (RHEA) Software and Patient Movement Throughout Health Care Facilities in Orange County **eFigure 2.** Network Map of Total Patient Sharing Among All Health Care Facilities in Orange County, California, Transferring on Average at Least 1 Patient per Week to Another Facility eReferences
