# Supplementary material for: Modeling Interventions to Reduce the Spread of Multidrug-Resistant Organisms Between Health Care Facilities in a Region
Source: JAMA Netw Open. 2021 Aug 4;4(8):e2119212. doi: 10.1001/jamanetworkopen.2021.19212 (PMC8339938; doi:10.1001/jamanetworkopen.2021.19212)
Supplement: Supplement. — eMethods. Description of Orange County, RHEA OC Software, and Model and Simulations eTable 1. MRSA and CRE Model Input Parameters, Values, and Sources eTable 2. Mean Number of New MRSA Carriers Over Time eTable 3. Mean Number of New CRE Carriers Over Time eFigure 1. Overview of the Structure of the Regional Healthcare Ecosystem Analyst (RHEA) Software and Patient Movement Throughout Health Care Facilities in Orange County eFigure 2. Network Map of Total Patient Sharing Among All Health Care Facilities in Orange County, California, Transferring on Average at Least 1 Patient per Week to Another Facility eReferences [file jamanetwopen-e2119212-s001.pdf]

Supplementary Online Content

Bartsch SM, Wong KF, Mueller LE, et al. Modeling interventions to reduce the spread of multidrug-resistant organisms between health care facilities in a region. *JAMA Netw Open*. 2021;4(8):e2119212. doi:10.1001/jamanetworkopen.2021.19212

eMethods. Description of Orange County, RHEA OC Software, and Model and Simulations

eTable 1. MRSA and CRE Model Input Parameters, Values, and Sources

eTable 2. Mean Number of New MRSA Carriers Over Time

eTable 3. Mean Number of New CRE Carriers Over Time

eFigure 1. Overview of the Structure of the Regional Healthcare Ecosystem Analyst (RHEA) Software and Patient Movement Throughout Health Care Facilities in Orange County

eFigure 2. Network Map of Total Patient Sharing Among All Health Care Facilities in Orange County, California, Transferring on Average at Least 1 Patient per Week to Another Facility

eReferences

This supplementary material has been provided by the authors to give readers additional information about their work.

© 2021 Bartsch SM et al. *JAMA Network Open*.

eMethods Description of Orange County, RHEA OC Software, and Model and Simulations

Orange County (OC), California

OC is the sixth largest US county with 3.2 million residents and 102 healthcare facilities serving adult patients: 28 acute care facilities (including 5 long-term acute care hospitals, LTACHs) and 74 nursing homes. OC serves, on average, 240,955 hospital admissions, 705 LTACH admissions, and 36,245 NH residents annually. On average, these facilities have reported 557 incident MRSA infections and 219 incident CRE infections, each year based on clinical cultures (unpublished data).

RHEA: OC Software

We used our custom-designed software, the Regional Healthcare Ecosystem Analyst (RHEA)[1,2], to generate an agent-based model (ABM) of OC, California and to simulate the spread of MRSA and CRE and evaluate the impact of various potential infection prevention interventions. RHEA: OC included detailed representations of all 102 healthcare facilities and the patients moving among these facilities and the community[1-9] and was populated based upon detailed line-item hospitalization and nursing home data.[10,11] Patient movement between and among the various types of healthcare facilities and the community has been previously described.[1-9]

Model and Simulations

eFigure 1 illustrates how patients move in the model. Briefly, in RHEA, each patient is represented by a virtual computational agent and could be a carrier or non-carrier for a specified pathogen. Each simulated day, these agents move from the community or other healthcare facility into the various healthcare facilities in OC. Each virtual healthcare facility has a number of beds (based on its actual bed count). Hospitals consist of multiple units [general

© 2021 Bartsch SM et al. *JAMA Network Open*.

units and intensive care units (ICUs)] with patient mixing within units, while nursing homes allow equal mixing throughout the facility, representing the high degree of social interactions among residents. Once a patient is admitted to a facility, a probability draw determines which of that facility's wards/units the patient enters, and a draw from a facility- and unit-specific length-of-stay (LOS) distribution determines how long the patient will remain in the ward/unit and facility. Patients colonized with a pathogen draw from a pathogen- and facility-specific LOS distribution to determine their LOS. Each day, within each ward/unit, patients mix homogeneously, and pathogen carriers can transmit to non-carriers, based on a ward- and facility-specific transmission coefficient (beta, β). Each day the number of new MDRO-carriers in each unit = β*susceptible patients in that unit*infectious patients in that unit. Once the patient's LOS elapses, the patient leaves the facility and has probabilities of returning to the community, directly transferring to another OC facility, or returning to the community for a period of time before being re-admitted to the same or another facility. Upon discharge, pathogen carriers have an increased risk of readmission within 365 days of discharge compared to non-carriers. Upon transfer to a hospital, 50% of LTACH patients and 20% of nursing home residents[12] are assigned to an ICU, representing those patients who require mechanical respiratory ventilation or other forms of intensive care. Additionally, during a nursing home stay, residents could experience a brief hospitalization during which his/her bed was held (i.e., temporary discharge). eTable 1 lists the model input parameters, values, and sources.

When simulating MRSA transmission, on any given day each patient could be either an MRSA carrier or non-carrier. MRSA carriers draw from facility-specific MRSA LOS distributions that are longer than the distribution for non-MRSA carriers (average 5.5 days longer

© 2021 Bartsch SM et al. *JAMA Network Open*.

countywide).[10] MRSA carriage was deemed to be persistent for one-third of carriers[13], while the remaining two-thirds experienced a linear spontaneous loss (25% over 274 days after initial colonization[14]). Our initial conditions assume an endemic MRSA prevalence in each facility (eTable 1) based on data in OC healthcare facilities.[15,16] We assumed a certain influx of MRSA from the community (e.g., admissions from the community). For hospitals, this influx was optimized such that the model targeted facility-specific point prevalence data; for nursing homes, it was set to 10%.[17] We parameterized facility-specific and unit-specific transmission coefficients (betas) to provide the MRSA target incidence for each specific facility and unit type (eTable 1). While these coefficients remained the same through the simulated period, they accounted for differences in MRSA susceptibility among patients in different facilities and transmission through staff members. Our model did not have separate betas for patients with active MRSA infections vs. asymptomatic carriers based on evidence that suggest no difference.[18] Simulations with these transmission coefficients resulted in MRSA prevalence values in simulated year 2017 that accurately reflected real-world observations, specifically: a model-generated 8.3% prevalence across all hospitals (6.4%-17.9% in individual hospitals) compared to a 7% prevalence[15]; a model-generated 28.7% prevalence across all LTACHs (19.7%-34.6% in individual LTACHs) compared to a median 30% and average 33% MRSA prevalence; and a model-generated 41.5% prevalence across all nursing homes (3.2%-45.8% in individual nursing homes) compared to a 42% prevalence.

When simulating CRE transmission, on any given day each patient could be either a CRE carrier or non-carrier. CRE carriers drew from a facility-specific CRE LOS distribution (on average 7.6 days longer than non-carriers) estimated using VRE-carrier data in OC.[10] Upon discharge,

CRE carriers had a 1.8-fold increased risk of readmission within 365 days of discharge compared to non-CRE carriers. Our initial conditions assumed there were no CRE cases in the healthcare facilities nor in recently discharged patients at day 0. Again, we parameterized facility- and ward-specific CRE transmission coefficients (beta), which were held throughout the simulation. Each nursing home and LTACH's beta was parameterized to reach a target prevalence of 10% in LTACHs and 3% in nursing homes 5 years from CRE introduction (year 0; eTable 1). We then parameterized the ICU and general unit betas, taking actual OC data into account, so that CRE prevalence trends matched those currently seen in OC facilities based upon epidemiologic surveys conducted in year 4 of CRE emergence.[19] The betas for ICUs and general units were 75% and 50% of the average nursing home betas, respectively. CRE carriage was deemed to be persistent for 30% of carriers[20,21], while the remaining 70% experienced a sigmoidal spontaneous loss with 45% loss at 12 months.[22] Simulations with these transmission coefficients resulted in CRE prevalence values in simulated year 4 and 5 that accurately reflected real-world observations, specifically: a model-generated 0.96% prevalence across all hospitals (0.3%-2.8% in individual hospitals) in year 4, which followed observed OC facility specific data[19] and 1.2% (0.4%-3.7% in individual hospitals) in year 7 compared to a 2% prevalence among only those patients on contact precautions from a prevalence survey conducted in 2017[15]; a model-generated 14.3% prevalence across all LTACHs (10.6%-17.8% in individual LTACHs) in year 5 compared to 10% target prevalence at year 5 and an 8% surveyed prevalence[16]; and a model-generated 3.7% prevalence across all nursing homes (0.03%-4.1% in individual nursing homes) in year 5 compared to a 3% prevalence[16].

© 2021 Bartsch SM et al. *JAMA Network Open*.

We also compared the resulting simulated reduction in MRSA prevalence when using the probabilities of being successfully decolonized based on clinical trials to the MRSA prevalence reductions reported in different decolonization clinical trials not reporting the probability of clearance. When simulating CHG bathing alone, an 18% probability of clearance[23] resulted in an median 9.5% (range: 2.4%-17.2%) reduction in MRSA prevalence in individual hospital ICUs, which is in line with the 14.6% reduction found in clinical trials, with a baseline compliance of 65%[24,25]. When simulating a decolonization regimen consisting of CHG bathing plus nasal product for MRSA, a 39%, 27%, and 3% probability of clearance for the decolonization regimen in hospitals, LTACHs, and nursing homes[26-30], respectively, resulted in a median 14.8% (range: 5.1%-14.7%) reduction in MRSA prevalence in individual hospital ICUs after 6 months, 20.2%-42.7% reduction in LTACHs after 6 months, and median 18.1% (range: 9.7%-21.1%) reduction in individual nursing homes after 3 months, which is low compared to the 37% reduction in MRSA prevalence in hospitals and LTACHs (assuming 80% compliance)[24] and a 30% reduction in nursing homes (assuming 80% CHG compliance and 60% iodophor compliance)[31]. These differences can be accounted for by different methods and patient populations in the various clinical trials. Thus, we varied these probabilities of clearance widely in sensitivity analyses.

© 2021 Bartsch SM et al. *JAMA Network Open*.

eTable 1. MRSA and CRE Model Input Parameters, Values, and Sources

Parameter	Value	Reference
**MRSA Parameters**		
MRSA prevalence (target at baseline)		
In hospitals	7%	[15]
In long-term acute care hospitals (LTACHs)	33%	[16]
In nursing homes	42%	[16]
MRSA incidence per admission length of stay		
In general units	0.01	[1]
In intensive care units (ICUs)	0.03	[4]
In LTACHs	0.10	[17]
In nursing homes[a]	0.20 (0.12)	[17]
Persistent MRSA carriers	33%	[13]
Spontaneous loss for MRSA	25% over 274 days	[14]
**Intervention Parameters**		
Active surveillance cultures[b]		
Sensitivity	75%	[32-35]
Specificity	97.1%	[36]
Turnaround time (days)	2	[36]
Contact precaution effectiveness[c]	40%	[37-41]
Percent on contact precautions for other reasons (other than MRSA)		Expert Opinion; Unpublished Data
In hospitals	2.7%	
In LTACHs	35.5%	
In nursing homes	1.3%	
Inter-facility communication for known patient MRSA status	50%	Expert Opinion[42,43]
Decolonization		
Probability of successful clearance with CHG-only bathing	18%	[23]
Probability of successful clearance with CHG bathing plus iodophor		
In hospitals	39%	[26-30]
In LTACHs	27%	[26]
In nursing homes	3%	Assumption[d]
Relapse after 90 days (linear over time)	20%	[14,27]
Relapse after 240 days (linear over time)	32%	[14,27,44]
**CRE Parameters**		
Point prevalence in LTACH (for year 5)	10%	Expert Opinion
Point prevalence in nursing homes (for year 5)	3%	[16]
Ratio of carriers to clinical isolates (iceberg effect)	8:1	[19,45,46e]
Increased risk of readmission for CRE carriers (on discharge)	80%	[47f]
Persistent carriers (remain colonized)	30%	[20,21]
Spontaneous loss for CRE	45% at 12 months	[22]

© 2021 Bartsch SM et al. *JAMA Network Open*.

**Intervention Parameters**		
Contact precautions effectiveness[c]	40%	[37-41]
Percent on contact precautions for other reasons (other than CRE)		Expert Opinion; Unpublished Data
In hospitals	9.0%	
In LTACHs	52.4%	
In nursing homes	2.0%	
Inter-facility communication for known patient CRE status	50%	Expert Opinion[42,43]
CHG bathing effectiveness[c]		
Prior to SHIELD intervention	39%	[46h]
With SHIELD intervention[g]	48%	[46]
Contact precautions plus CHG bathing effectiveness		
Prior to SHIELD intervention	48%	Assumption
With SHIELD intervention[g]	60%	Assumption

NOTE: LTACH: long-term acute care hospitals; MRSA: methicillin-resistant *Staphylococcus aureus*; CRE: carbapenem-resistant *Enterobacteriaceae*; CHG: chlorhexidine gluconate

[a]Values derived; mean (standard deviation) across all facilities with that type of ward; values were facility-specific

[b]Sensitivity and specificity of the active surveillance cultures accounts for swabbing being done incorrectly or not done on multiple bodies sites

[c]Combination of intervention efficacy and staff compliance with and proper application/use of intervention

[d]Lower value in nursing homes due to differences in the quality and frequency of bathing (e.g., lower staff/resident ration, bathing 3 days/week) and resident characteristics that make it more difficult to clear colonization (e.g., refusals/confusion, comorbidities, indwelling devices, wounds, bed bound status).[48]

[e]And personal communication with Michael Lin MD, Rush University

[f]And personal communication with Dawn Terashita MD, Los Angeles County Department of Health

[g]Assumes higher staff compliance with and application/use of intervention

[h]Adjusted for lower compliance

© 2021 Bartsch SM et al. *JAMA Network Open*.

eTable 2. Mean Number of New MRSA Carriers Over Time

	Time After SHIELD Start					
	**0.5 years**	**1 years**	**1.5 years**	**2 years**	**2.5 years**	**3 years**
**Number of New MRSA Carriers in Target (Participating) Facilities - Direct Impact**						
Continued existing measures	2,693.8 (1.8)	5,837.3 (3.0)	8,532.2 (4.0)	11,226.7 (5.2)	13,920.4 (6.3)	16,165.8 (6.8)
Increasing contact precaution effectiveness to 48%	2,657.9 (2.4)	5,750.3 (3.0)	8,399.6 (4.1)	11,049.4 (5.3)	13,698.3 (6.0)	15,905.4 (6.9)
Increasing contact precaution effectiveness to 64%	2,592.7 (2.7)	5,593.4 (3.6)	8,161.5 (4.2)	10,728.5 (4.8)	13,295.1 (5.3)	15,433.6 (5.6)
Increasing inter-facility communication to 60%*	2,693.2 (1.8)	5,835.0 (3.0)	8,529.4 (4.0)	11,224.1 (4.7)	13,918.8 (4.9)	16,164.0 (5.9)
Increasing inter-facility communication to 80%*	2,693.1 (2.2)	5,836.4 (3.8)	8,530.7 (4.7)	11,225.0 (5.8)	13,919.4 (6.0)	16,163.9 (6.6)
Decolonization with a clearance probability of 24% in hospitals, 18% in LTACHs, and 1% in NHs	2,622.6 (2.0)	5,563.6 (3.3)	8,034.4 (3.8)	10,473.8 (4.4)	12,890.2 (5.3)	14,890.1 (6.1)
Decolonization with a clearance probability of 39% in hospitals, 27% in LTACHs, and 3% in NHs	2,502.5 (2.7)	5,102.5 (4.0)	7,199.5 (5.1)	9,216.8 (5.9)	11,174.8 (7.4)	12,769.8 (8.8)
Decolonization with a clearance probability of 54% in hospitals, 42% in LTACHs, and 16.7% in NHs	2,029.5 (2.1)	3,684.3 (3.6)	4,903.0 (5.4)	6,000.5 (6.8)	7,014.0 (7.6)	7,812.1 (7.8)
**Number of New MRSA Carriers in Non-Target (Non-Participating) Facilities - Indirect Impact**						
Continued existing measures	2,201.6 (1.7)	4,768.7 (3.2)	6,969.2 (3.7)	9,168.8 (4.4)	11,368.3 (4.9)	13,200.6 (5.4)
Increasing contact precaution effectiveness to 48%	2,200.0 (1.9)	4,766.5 (3.4)	6,965.4 (3.9)	9,163.4 (4.1)	11,360.6 (4.4)	13,191.5 (4.1)
Increasing contact precaution effectiveness to 64%	2,198.1 (1.7)	4,760.4 (2.7)	6,955.7 (3.3)	9,149.6 (4.3)	11,344.1 (4.8)	13,172.2 (5.7)
Increasing inter-facility communication to 60%*	2,201.0 (1.8)	4,769.3 (2.8)	6,969.6 (3.8)†	9,169.6 (4.5)†	11,368.7 (5.2)†	13,201.8 (5.3)†
Increasing inter-facility communication to 80%*	2,200.8 (1.6)	4,768.3 (2.8)	6,968.4 (2.8)	9,167.9 (3.7)	11,366.6 (4.7)	13,199.2 (4.9)
Decolonization with a clearance probability of 24% in hospitals, 18% in LTACHs, and 1% in NHs	2,199.3 (1.3)	4,760.5 (2.1)	6,952.5 (2.9)	9,141.7 (3.2)	11,328.9 (4.2)	13,150.0 (4.9)
Decolonization with a clearance probability of 39% in hospitals, 27% in LTACHs, and 3% in NHs	2,197.0 (1.7)	4,748.8 (2.8)	6,929.2 (4.2)	9,103.8 (4.7)	11,275.0 (5.1)	13,081.8 (5.4)
Decolonization with a clearance probability of 54% in hospitals, 42% in LTACHs, and 16.7% in NHs	2,189.7 (1.4)	4,717.9 (2.9)	6,873.6 (3.1)	9,022.1 (3.7)	11,164.1 (4.0)	12,945.5 (4.8)

*Only applies to inter-facility transfers. If patient is readmitted to the same facility, this information is preserved and contact precautions are applied 100% of the time

†Values not substantially higher than continuing current control measures and are due to model variation

© 2021 Bartsch SM et al. *JAMA Network Open*.

eTable 3. Mean Number of New CRE Carriers Over Time

	Time After SHIELD Start					
	**0.5 years**	**1.0 years**	**1.5 years**	**2.0 years**	**2.5 years**	**3.0 years**
**Number of New CRE Carriers in Target (Participating) Facilities - Direct Impact**						
Continued existing measures	288.2 (1.8)	637.0 (3.7)	944.6 (5.1)	1,207.1 (6.1)	1,584.6 (7.7)	1,917.2 (8.8)
Increasing contact precautions effectiveness to 48%	281.8 (1.9)	620.3 (4.0)	918.0 (5.5)	1,171.4 (6.7)	1,535.2 (8.6)	1,855.6 (9.5)
Increasing contact precautions effectiveness to 64%	271.1 (2.3)	593.9 (4.0)	876.6 (5.5)	1,117.1 (6.6)	1,461.2 (8.3)	1,764.3 (9.7)
Increasing inter-facility communication to 60%*	288.9 (2.3)†	638.0 (4.6)†	946.2 (6.4)†	1,209.2 (7.7)†	1,586.9 (9.4)†	1,920.1 (11.0)†
Increasing inter-facility communication to 80%*	288.1 (1.6)	636.0 (3.2)	943.3 (4.9)	1,206.1 (6.3)	1,583.6 (7.9)	1,915.9 (9.2)
CHG bating effectiveness 42%	188.9 (1.1)	374.8 (2.5)	517.5 (3.2)	628.5 (4.1)	775.9 (4.8)	897.0 (5.3)
CHG bating effectiveness 48%	153.8 (1.0)	285.6 (1.8)	377.7 (2.6)	445.1 (3.1)	530.2 (3.7)	597.0 (4.2)
CHG bating effectiveness 54%	124.7 (1.1)	219.3 (1.9)	280.6 (2.3)	323.6 (2.8)	376.1 (2.3)	416.4 (2.8)
**Number of New CRE Carriers in Non-Target (Non-Participating) Facilities - Indirect Impact**						
Continued existing measures	251.0 (1.8)	556.8 (3.7)	829.4 (5.0)	1,063.3 (6.3)	1,400.9 (7.8)	1,699.4 (9.1)
Increasing contact precautions effectiveness to 48%	251.4 (1.8)	556.9 (3.6)	829.0 (4.8)	1,062.4 (5.8)	1,399.1 (7.1)	1,696.8 (8.2)
Increasing contact precautions effectiveness to 64%	250.6 (1.2)	555.3 (2.4)	826.2 (3.2)	1,058.1 (3.9)	1,391.9 (4.7)	1,686.5 (5.4)
Increasing inter-facility communication to 60%*	251.0 (1.7)	556.9 (3.7)†	829.8 (5.3)†	1,064.0 (6.2)†	1,401.5 (7.9)†	1,699.9 (9.4)†
Increasing inter-facility communication to 80%*	251.0 (1.6)	556.6 (3.1)	828.8 (4.4)	1,062.6 (5.8)	1,399.5 (7.4)	1,697.8 (8.7)
CHG bating effectiveness 42%	249.8 (1.6)	550.0 (3.6)	813.5 (5.2)	1,036.6 (6.5)	1,354.6 (8.2)	1,632.3 (9.5)
CHG bating effectiveness 48%	248.9 (1.3)	544.9 (2.8)	801.9 (4.4)	1,017.4 (5.5)	1,321.7 (7.0)	1,585.0 (8.4)
CHG bating effectiveness 54%	248.4 (1.6)	541.7 (2.8)	794.6 (3.8)	1,005.6 (4.4)	1,301.7 (3.8)	1,556.6 (4.4)

*Only applies to inter-facility transfers. If patient is readmitted to the same facility, this information is preserved and contact precautions are applied 100% of the time.
†Values not substantially higher than continuing current control measures and are due to model variation.

© 2021 Bartsch SM et al. *JAMA Network Open*.

eFigure 1. Overview of the Structure of the Regional Healthcare Ecosystem Analyst (RHEA) Software and Patient Movement Throughout the Health Care Facilities in Orange County

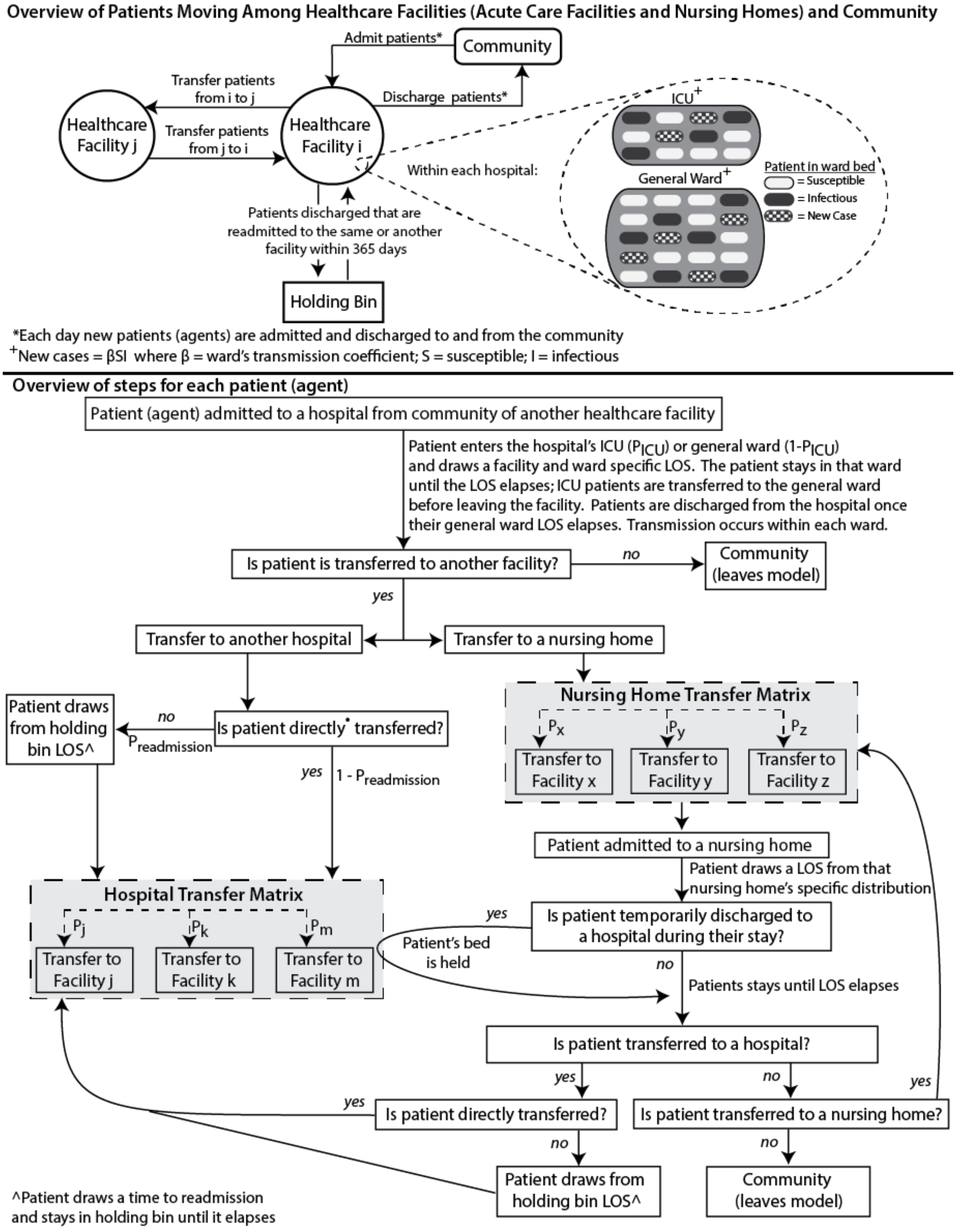

© 2021 Bartsch SM et al. *JAMA Network Open*.

eFigure 2. Network Map of Total Patient Sharing Among All Health Care Facilities in Orange County, California, That Transferred on Average at Least 1 Patient per Week to Another Facility (With or Without an Intervening Stay in the Community; n = 89)

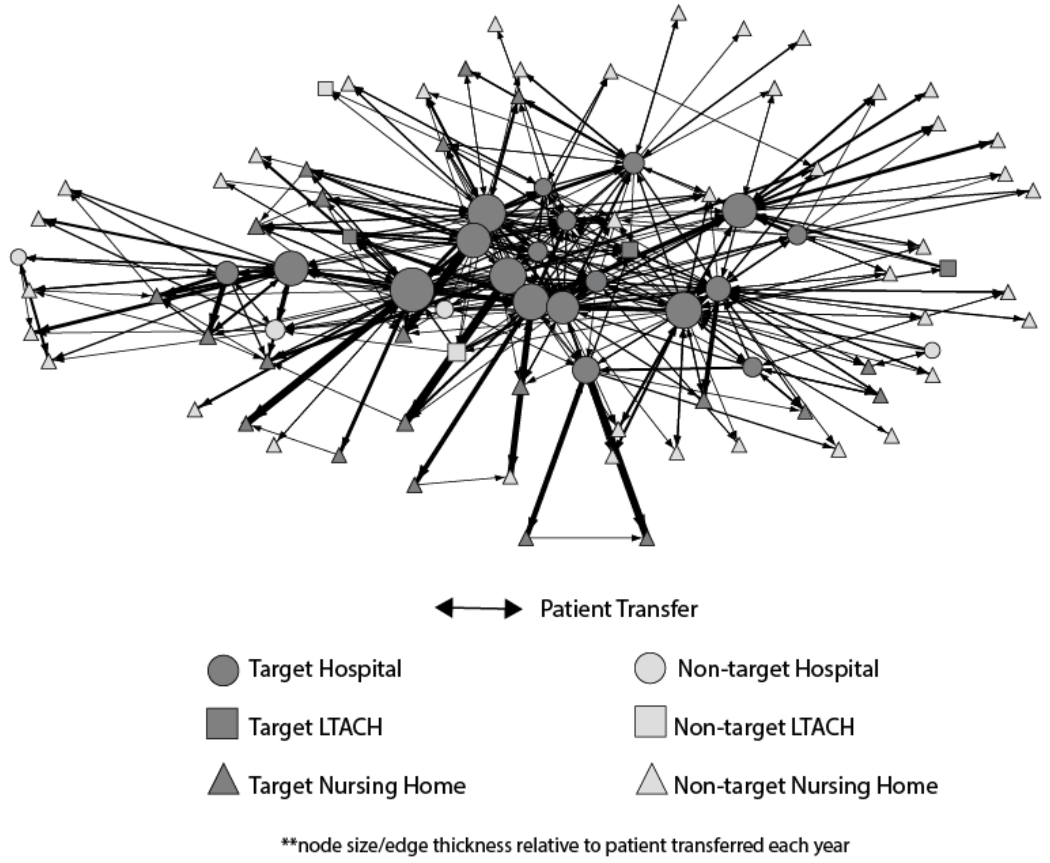

Edges (i.e., directed lines) are weighted by the number of patients transferred in a year.

© 2021 Bartsch SM et al. *JAMA Network Open*.

eReferences

1. Lee BY, McGlone SM, Wong KF, et al. Modeling the spread of methicillin-resistant *Staphylococcus aureus* (MRSA) outbreaks throughout the hospitals in Orange County, California. *Infect Control Hosp Epidemiol.* 2011;32(6):562-572.
2. Lee BY, Wong KF, Bartsch SM, et al. The Regional Healthcare Ecosystem Analyst (RHEA): simulation modeling tool to assist infectious disease control in a health system. *J Am Med Inform Assoc.* 2013;20(e1):e139-146.
3. Lee BY, Bartsch SM, Wong KF, et al. The importance of nursing homes in the spread of methicillin-resistant *Staphylococcus aureus* (MRSA) among hospitals. *Med Care.* 2013;51(3):205-215.
4. Huang SS, Avery TR, Song Y, et al. Quantifying interhospital patient sharing as a mechanism for infectious disease spread. *Infect Control Hosp Epidemiol.* 2010;31(11):1160-1169.
5. Lee BY, Bartsch SM, Wong KF, et al. Simulation shows hospitals that cooperate on infection control obtain better results than hospitals acting alone. *Health Aff.* 2012;31(10):2295-2303.
6. Lee BY, Yilmaz SL, Wong KF, et al. Modeling the regional spread and control of vancomycin-resistant enterococci (VRE). *Am J Infect Control.* 2013;41(8):668-673.
7. Lee BY, Singh A, Bartsch SM, et al. The potential regional impact of contact precuation use in nursing homes to control methicillin-resistant *Staphylococcus aureus*. *Infect Control Hosp Epidemiol.* 2013;34(2):151-160.
8. Lee BY, Bartsch SM, Wong KF, et al. The potential trajectory of carbapenem-resistant *Enterobacteriaceae*, an emerging threat to health-care facilities, and the impact of the Centers for Disease Control and Prevention toolkit. *Am J Epidemiol.* 2016;183(5):471-479.
9. Lee BY, Bartsch SM, Wong KF, et al. Beyond the Intensive Care Unit (ICU): Countywide Impact of Universal ICU Staphylococcus aureus Decolonization. *Am J Epidemiol.* 2016;183(5):480-489.
10. Office of Statewide Health Planning and Development. California Inpatient Data Reporting Manual, Medical Information Reporting for California, Seventh Edition, Version 8.3. http://www.oshpd.ca.gov/HID/MIRCal/Text_pdfs/ManualsGuides/IPManual/TofC.pdf. Published 2014. Updated September 2014. Accessed June 16, 2014.
11. Centers for Medicare & Medicaid Services. Long Term Care Minimum Data Set. Centers for Medicare & Medicaid Services. Published 2014. Accessed June 2014.
12. Wang HE, Shah MN, Allman RM, Kilgore M. Emergency department visits by nursing home residents in the United States. *Journal of the American Geriatrics Society.* 2011;59(10):1864-1872.
13. Kluytman J, van Belkum A, Verbrugh H. Nasal carriage of *Staphylococcus aureus*: epidemiology, underlying mechanisms, and associated risks. *Clinical Microbiology Reviews.* 1997;10(3):505-520.
14. Huang SS, Singh RD, Eells SJ, et al. Impact of post-discharge chlorhexidine (CHG) and mupirocin on MRSA carriage in a randomized trial. ID Week (2nd Annual Joint Meeting of ISDA, SHEA, HIVMA, and PIDS); October 2-6, 2013, 2013; San Francisco, CA.

© 2021 Bartsch SM et al. *JAMA Network Open*.

15. McKinnell JA, Miller LG, Eells SJ, Cui E, Huang SS. A systematic literature review and meta-analysis of factors associated with methicillin-resistant *Staphylococcus aureus* colonization at time of hospital or intensive care unit admission. *Infection Control and Hospital Epidemiology.* 2013;34(10):1077-1086.
16. McKinnell JA, Singh RD, Miller LG, et al. The SHIELD Orange County Project -Multi Drug-Resistant Organism (MDRO) Prevalence in 21 Nursing Homes and Long Term Acute Care Facilities in Southern California. *Clin Infect Dis.* 2019.
17. Murphy CR, Quan V, Kim D, et al. Nursing home characteristics associated with methicillin-resistant *Staphylococcus aureus* (MRSA) burden and transmission. *BMC Infectious Disease.* 2012;12:269.
18. Chang S, Sethi AK, Eckstein BC, Stiefel U, Cadnum JL, Donskey CJ. Skin and environmental contamination with methicillin-resistant Staphylococcus aureus among carriers identified clinically versus through active surveillance. *Clin Infect Dis.* 2009;48(10):1423-1428.
19. Gohil S, Singh RD, Gombosev A, et al. Emergence of Carbapenemase Resistant Enterobactereaceae (CRE) in Orange County, CA and Support for Regional Strategies to Limit Spread. Paper presented at: ID Week: A Joint Meeting of the Infectious Disease Society of America, Society for Healthcare Epidemiology of America, HIV Medicine Association, and the Pediatric Infectious Disease Society; October 8-12, 2014; Philadelphia, PA.
20. O'Fallon E, Gautam S, D'Agata EMC. Colonization with multidrug-resistant gram-negative bacteria; prolonged duration and frequent co-colonization. *Clin Infect Dis.* 2009;48(10):1375-1381.
21. Feldman N, Adler A, Molshatzki N, et al. Gastrointestinal colonization by KPC-producing *Klebsiella pneumoniae* following hospital discharge: duration of carriage and risk factors for persistent carriage. *Clin Microbiol Infect.* 2013;19(1E190-196).
22. Bart Y, Paul M, Eluk O, Geffen Y, Rabino G, Hussein K. Risk Factors for Recurrence of Carbapenem-Resistant Enterobacteriaceae Carriage: Case-Control Study. *Infect Control Hosp Epidemiol.* 2015;36(8):936-941.
23. Harbarth S, Dharan S, Liassine N, Herrault P, Auckenthaler R, Pittet D. Randomized, placebo-controlled, double-blind trial to evaluate the efficacy of mupirocin for eradicating carriage of methicillin-resistant Staphylococcus aureus. *Antimicrob Agents Chemother.* 1999;43(6):1412-1416.
24. Huang SS, Septimus E, Kleinman K, et al. Targeted versus universal decolonization to prevent ICU infection. *New England Journal of Medicine.* 2013;368(24):2255-2265.
25. Climo MW, Yokoe DS, Warren DK, et al. Effect of daily chlorhexidine bathing on hospital-aquired infection. *New England Journal of Medicine.* 2013;368:533-542.
26. Camus C, Sebille V, Legras A, et al. Mupirocin/chlorexidine to prevent methicillin-resistant Staphylococcus aureus infections: post hoc analysis of a placebo-controlled, randomized trial using mupirocin/chlorhexidine and polymyxin/tobramycin for the prevention of acquired infections in intubated patients. *Infection.* 2014;42(3):493-502.
27. Simor AE, Phillips E, McGeer A, et al. Randomized controlled trial of chlorhexidine gluconate for washing, intranasal mupirocin, and rifampin and doxycycline versus no treatment for the eradication of methicillin-resistant *Staphylococcus aureus* colonization. *Clin Infect Dis.* 2007;44(2):178-185.

© 2021 Bartsch SM et al. *JAMA Network Open*.

28. Sai N, Laurent C, Strale H, Denis O, Byl B. Efficacy of the decolonization of methicillin-resistant Staphylococcus aureus carriers in clinical practice. *Antimicrob Resist Infect Control.* 2015;4:56.
29. Kline SE, Neaton JD, Lynfield R, et al. Randomized controlled trial of a self-administered five-day antiseptic bundle versus usual disinfectant soap showers for preoperative eradication of Staphylococcus aureus colonization. *Infect Control Hosp Epidemiol.* 2018;39(9):1049-1057.
30. Huang SS, Singh RD, Eells SJ, et al. Impact of post-discharge chlorhexidine (CHG) and mupirocin on MRSA carriage in a randomized trial. ID Week (2nd Annual Joint Meeting of ISDA, SHEA, HIVMA, and PIDS); October 2-6, 2013; San Francisco, CA.
31. Miller LG, McKinnell JA, Singh R, et al. Reduction of MDRO colonization in nursing home residents with routine use of chlorhexidine bathing and nasal iodophor (Project PROTECT). ID Week; October 26-30, 2016; New Orleans, LA.
32. Yang E, Tan J, Eells S, Rieg G, Tagudar G, Miller L. Body site colonization in patients with community-associated methicillin-resistant *Staphylococcus aureus* and other types of S. aureus skin infections. *Clinical Microbiology and Infection.* 2010 16(5):425-431.
33. Widmer AF, Mertz D, Frei R. Necessity of screening of both the nose and the throat to detect methicillin-resistant *Staphylococcus aureus* colonization in patients upon admission to an intensive care unit. *Journal of Clinical Microbiology.* 2008;46:835.
34. Rohr U, Wilhelm M, Muhr G, Gatermann S. Qualitative and semiquantitative characterization of nasal and skin methicillin-resistant *Staphylococcus aureus* carriage of hospitalized patients. *International Journal of Hygiene and Environmental Health.* 2004;207(1):51-55.
35. Mertz D, Frei R, Jaussi B, et al. Throat swabs are necessary to reliably detect carriers of *Staphyolcoccus aureus*. *Clinical Infectious Diseases.* 2007;2007(45):475-477.
36. Reyes J, Hidalgo M, Diaz L, et al. Characterization of macrolide resistance in gram-positive cocci from Colombian hospitals: a countrywide surveillance. *International Journal of Infectious Diseases.* 2007;11(4):329-336.
37. Bearman GM, Marra AR, Sessler CN, et al. A controlled trial of universal gloving versus contact precautions for preventing the transmission of multidrug-resistant organisms. *Am J Infect Control.* 2007;35(10):650-655.
38. Clock SA, Cohen B, Behta M, Ross B, Larson EL. Contact precautions for multidrug-resistant organisms: Current recommendations and actual practice. *Am J Infect Control.* 2010;38(2):105-111.
39. Golan Y, Doron S, Griffith J, et al. The impact of gown-use requirement on hand hygiene compliance. *Clin Infect Dis.* 2006;42(3):370-376.
40. Weber DJ, Sickbert-Bennett EE, Brown VM, et al. Compliance with isolation precautions at a university hospital. *Infect Control Hosp Epidemiol.* 2007;28(3):358-361.
41. Cromer AL, Hutsell SO, Latham SC, et al. Impact of implementing a method of feedback and accountability related to contact precautions compliance. *Am J Infect Control.* 2004;32(8):451-455.
42. Kim J, Segreti J, Tomich A, Tongma C, Hayden MK, Lin MY. Surveillance and inter-facility communication for carbapenem-resistant *Enterobacteriaceae* (CRE). Paper presented at: Society for Healthcare Epidemiology of America; May 18-21, 2016; Atlanta, GA.

© 2021 Bartsch SM et al. *JAMA Network Open*.

43. Bartsch SM, Wong KF, Stokes-Cawley OJ, et al. Knowing More of the Iceberg: How Detecting a Greater Proportion of Carbapenem-Resistant Enterobacteriaceae (CRE) Carriers Impacts Transmission. *Journal of Infectious Diseases.* 2019.
44. Immerman I, Ramos NL, Katz GM, Hutzler LH, Phillips MS, Bosco JA, 3rd. The persistence of *Staphylococcus aureus* decolonization after mupirocin and topical chlorhexidine: implications for patients requiring multiple or delayed procedures. *J Arthroplasty.* 2012;27(6):870-876.
45. Pisney LM, Barron MA, Kassner E, Havens D, Madinger NE. Carbapenem-resistant enterobacteriaceae rectal screening during an outbreak of New Delhi metallo-*B*-lactamase producing *Klebsiella pneumoniae* at an acute care hospital. *Infect Control Hosp Epidemiol.* 2014;35(4):434-436.
46. Hayden MK, Lin MY, Lolans K, et al. Prevention of colonization and infection by *Klebsiella pneumoniae* carbapenemase-producing enterobacteriaceae in long-term acute-care hospitals. *Clin Infect Dis.* 2015;60(8):1153-1161.
47. Marquez P, Terashita D. Long-term acute care hospitals and carbapenem-resistant *Enterobacteriaceae*: a reservoir for transmission. *Clin Infect Dis.* 2013;57(9):1253-1255.
48. McKinnell JA, Miller LG, Singh RD, et al. High Prevalence of Multidrug-Resistant Organism Colonization in 28 Nursing Homes: An "Iceberg Effect". *J Am Med Dir Assoc.* 2020;21(12):1937-1943 e1932.

© 2021 Bartsch SM et al. *JAMA Network Open*.
